# Supplementary material for: Nutritional Education Is an Effective Tool in Improving Beverage Assortment in Nurseries in Poland
Source: Healthcare (Basel). 2021 Mar 3;9(3):274. doi: 10.3390/healthcare9030274 (PMC8002118; doi:10.3390/healthcare9030274)

Figure S1. The overview of the project Eating healthy, growing healthy. *DCCs – day care centers.

Source: Myszkowska-Ryciak, J.; Harton, A. Do preschools offer healthy beverages to children? A nationwide study in Poland. *Nutrients* **2017**. DOI: 10.3390/nu9111167

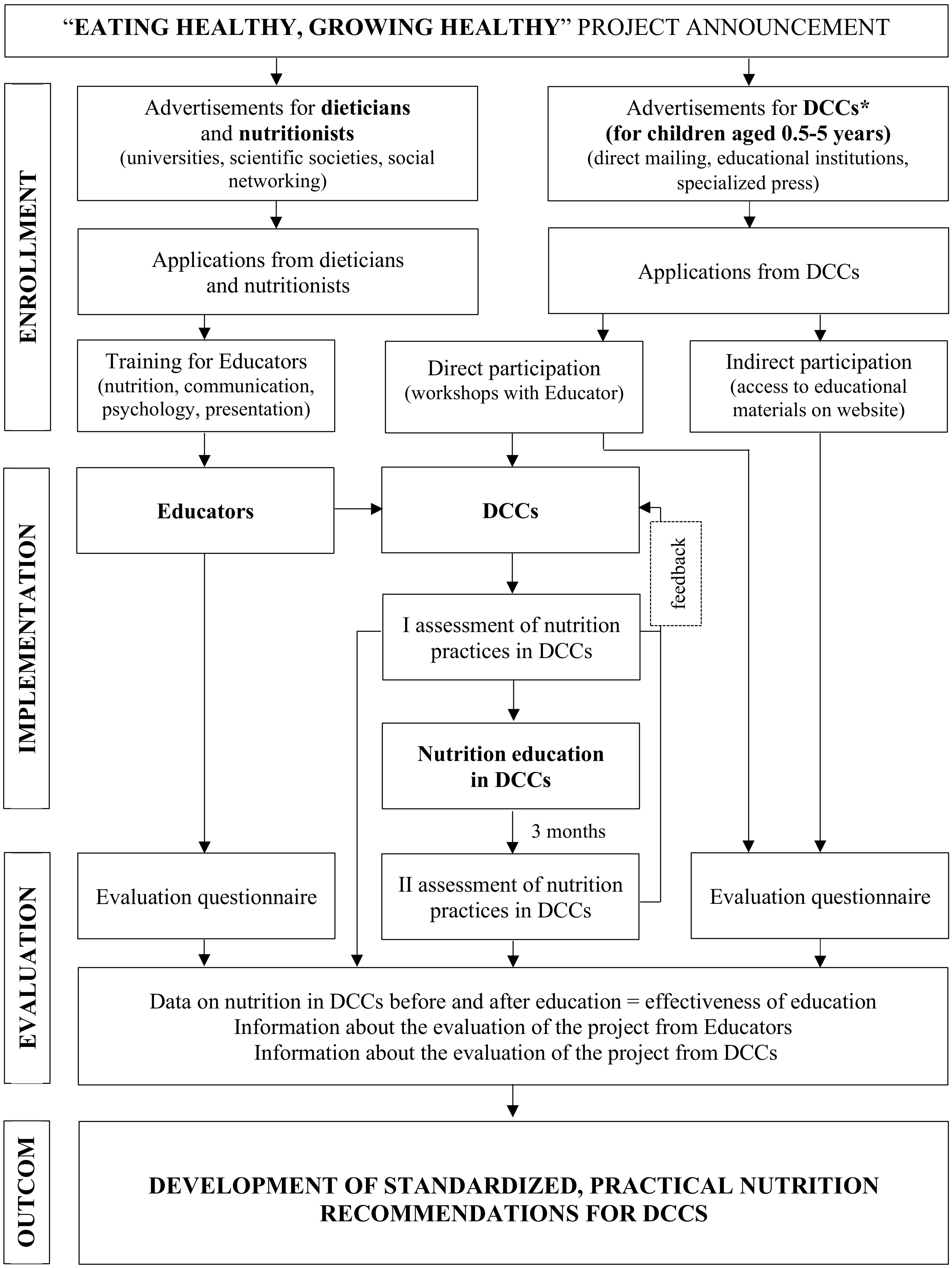

Supplement: Supplementary file 1 [file healthcare-09-00274-s001.pdf]
